# Carotenoid Profiling of Yellow-Flesh Peach Fruit

**DOI:** 10.3390/foods11121669

**Published:** 2022-06-07

**Authors:** Bintao Zhao, Meng Sun, Jiyao Li, Ziwen Su, Zhixiang Cai, Zhijun Shen, Ruijuan Ma, Juan Yan, Mingliang Yu

**Affiliations:** 1 Institute of Pomology, Jiangsu Academy of Agricultural Sciences/Jiangsu Key Laboratory for Horticultural Crop Genetic Improvement, No. 50 Zhongling Street, Nanjing 210014, China; zbt17730869939@163.com (B.Z.); sm183495665@163.com (M.S.); kaelthasli@gmail.com (J.L.); 2018804207@njau.edu.cn (Z.S.); czxly05@163.com (Z.C.); shenjaas@aliyun.com (Z.S.); marj311@163.com (R.M.); mly1008@aliyun.com (M.Y.); 2 College of Horticulture, Nanjing Agricultural University, No.1 Weigang, Nanjing 210095, China

**Keywords:** yellow-flesh peach, carotenoids, developmental period, indumentum, flesh color

## Abstract

In this study, the carotenoid profiles and content in 132 cultivars of yellow-flesh peach having different fruit developmental periods (short, middle, and long), fruit surface indumenta (glabrous and pubescent skin), and flesh colors (yellow, golden, and orange) were investigated. We simultaneously analyzed and compared the levels of five carotenoids (lutein, zeaxanthin, β-cryptoxanthin, α-carotene, and β-carotene) through high-performance liquid chromatography. Large differences in carotenoid content among germplasms were observed, with coefficients of variation ranging from 21.24% to 67.78%. The carotenoid content, from high to low, was as follows: β-carotene > zeaxanthin > α-carotene > β-cryptoxanthin > lutein. We screened several varieties with high carotenoid content, including zeaxanthin in ‘Ruiguang2’, β-cryptoxanthin in ‘NJN76’ and ‘TX4F244C’, and β-carotene and total carotenoids in ‘Jintong7’, ‘77-26-7’, and ‘77-20-5’. A longer fruit developmental period was associated with greater β-carotene accumulation but lowered the zeaxanthin and β-cryptoxanthin accumulation. The zeaxanthin, β-carotene, and total carotenoid concentrations significantly increased as the flesh color deepened, but the lutein and α-carotene levels remained similar among the three flesh colors. The classification index of the indumenta significantly affected the β-carotene and total carotenoid content (*p* < 0.05) and was higher in pubescent than glabrous skin.

## 1. Introduction

Yellow-flesh peaches, which are eaten fresh and as a canned, processed food [1,2], are a very important commercial peach (*Prunus persica* (L.)) type. Yellow-flesh peaches have various flesh colors, such as yellow, golden, and orange, and they contain various phytochemicals, such as soluble sugars, flavonoids, and carotenoids.

Containing conjugated double bonds that account for many fruit properties [3], the carotenoids give fruit and vegetables their bright yellow, orange, and red colors and are now recognized for their nutritional value [4]. They benefit human health in many ways. Some carotenoids, such as β-carotene, are precursors of vitamin A and consequently can be used to treat vitamin A deficiency [5,6]. In particular, lutein has many diverse effects. Capsanthin exhibits anti-tumor activity, attenuates obesity-induced inflammation [7,8], and increases plasma high-density lipoprotein levels [9]. Lutein and zeaxanthin decrease the risk of age-related macular degeneration and cancer [10,11]. β-Cryptoxanthin has a role in improving human health through bone homeostasis [12]. Thus, carotenoid levels and profiles are both crucial aspects of fruit quality [13].

Yellow-flesh peaches have rich germplasm resources and high economic and research values. Carotenoids in yellow-flesh peach have received attention because of their important physiological functions, and researchers began investigating them as early as 1970s and 1980s [14]. Breeders are interested in developing new yellow-flesh peach cultivars that are rich in carotenoids. Therefore, carotenoids have been intensively and widely studied. Researchers have examined methods for detecting the carotenoid components and concentrations [15,16] and the changes in these components as fruit develop [17,18,19,20], as well as the effects of cultivating methods [21,22,23], harvesting time [24], and storage conditions [25,26] on carotenoid concentrations and antioxidant capacity. Additionally, the mechanisms of carotenoid biosynthesis have been investigated [27,28,29]. However, limited comparative evaluations of carotenoid content have been performed in large numbers of germplasm resources [30], and no systematic analyses of the relationships between carotenoids and other fruit characteristics. In particular, no research has examined the fruit development period and fruit surface indumenta, which are very important descriptive indices, as is flesh color.

The main aim of the current work was first to determine the carotenoid content in 132 cultivars with different fruit developmental periods (short, middle, and long), fruit surface indumenta (glabrous and pubescent skin), and flesh colors (yellow, golden, and orange). In detail, we detected five carotenoids, lutein, zeaxanthin, β-cryptoxanthin, α-carotene, and β-carotene, which are the main known carotenoids in yellow-flesh peach [19]. Then, the differences in carotenoid concentrations among these different types of yellow-flesh peach were compared, and the relationships between carotenoids and fruit developmental period, surface indumenta, and flesh color were analyzed. This study aimed to identify differences in carotenoid contents among varieties and screen germplasms having high carotenoid contents.

## 2. Material and Methods

### 2.1. Plant Materials

**Figure 1:**
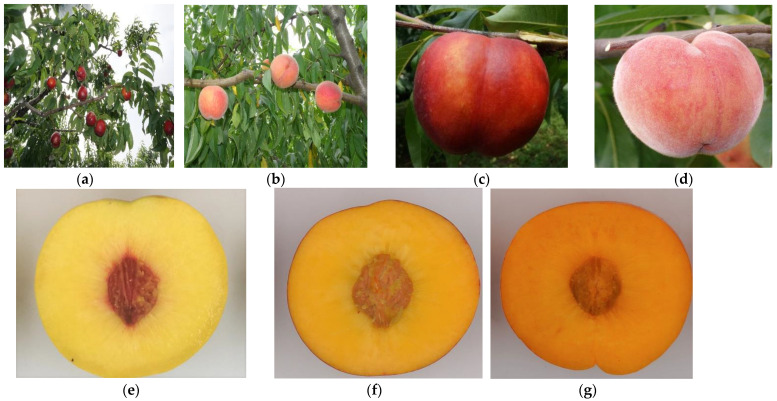
Studied materials of different peach types. (**a**) Peach with shortest fruit developmental period ‘Chaowuyuehuo’ (63 d); (**b**) peach with longest fruit developmental period ‘Shamenlaite’ (151 d); (**c**) peach with glabrous skin ‘Ao19’; (**d**) peach with pubescent skin ‘Beibeile’; (**e**) peach with yellow flesh color ‘Ruiguang2’; (**f**) peach with golden flesh color ‘Zijinhong2’; (**g**) peach with orange flesh color ‘TX4F244C’.

Fruit of 132 yellow-flesh peach cultivars was obtained from the National Peach Germplasm Repository (Nanjing, China, 32°2′ N, 118°52′ E, 11 m above sea level) in 2019, with each cultivar represented by two trees. The test cultivars were 5-year-old mature trees of Y shape, with a row spacing of 3 × 5 m, and the conventional cultivation measures were managed. Fruit samples of each cultivar were collected at maturity time from the two trees, with each sample consisting of 18 fruits with three replicates. The maturity time refers to the time when the fruit (especially the fruit bottom) peel loses its green background color. On the basis of the evaluation of peach trait descriptions and classifications in “Chinese fruit (Peach)” and “Descriptors and Data Standard for Peach (*Prunus persica* L.)” [1,2], 132 cultivars of yellow flesh peaches with different fruit developmental periods, fruit surface indumenta, and flesh colors, were investigated. The fruit developmental period is the number of days from flowering to fruit maturity. In this study, the yellow-flesh peach resources’ periods ranged from 63 to 151 days. By calculating the extremes, means, and standard deviations, the periods were classified as short (from 63 to 90 days, total 40), medium (from 91 to 120 days, total 59), and long (from 120 to 151 days, total 33). The peaches were divided into pubescent (total 88) and glabrous (total 44), and the peach flesh was divided into yellow (total 34), golden (total 66), and orange (total 32) according to pantone international color card (Figure 1 and Table S1). Fruits from each sample were mechanically peeled and cored, and the flesh was cut into small sections, which were then mixed and immediately frozen in liquid nitrogen and stored at −75 °C until used.

### 2.2. Carotenoid Extraction and Measurement

We extracted and measured five carotenoids in the fruit flesh of 132 yellow-flesh peach cultivars in accordance with the method of Yan et al. [16], with some modifications. Frozen fruit (1 g) was homogenized in 6 mL of high-performance liquid chromatography (HPLC)-grade acetone and extracted using ultrasonic-assisted extraction for 2 h in the dark. Then, the extract was centrifuged (10,000 g for 10 min at 4 °C) and passed through a 0.22-µm filter for analysis (Agilent 1260 Infinity HPLC system, Milford, MA, USA). Samples (20 µL of extract) were analyzed using a YMC-C30 column (4.6 × 250 mm, 5 μm) coupled with a diode array detector at 450 nm, with HPLC-grade methyl tertiary butyl ether and methanol (V:V = 30:70) as the solvent. The flow rate was 1.0 mL·min^−1^, and the temperature was 25 °C. Standards of lutein, zeaxanthin, β-cryptoxanthin, α-carotene, and β-carotene were purchased from Solarbio (Beijing Solarbio Science & Technology Co., Ltd., Beijing, China). Compounds were quantified by comparing the peak areas and are presented as mg per kg of fresh weight (mg·kg^−1^ FW). Samples were analyzed in three technical replicates.

### 2.3. Statistical Analysis

Quantitative data were analyzed in SPSS version 18.0 (SPSS Inc., Chicago, IL, USA). Normality analysis, one-way analysis of variance, and correlation analysis were performed. Independent samples t-tests were used to compare carotenoid concentrations among peaches with different flesh colors and between fruits with different fruit surface indumenta. The level of significance was set at *p* < 0.05.

## 3. Results

### 3.1. Carotenoids in Yellow-Flesh Peach

**Figure 2:**
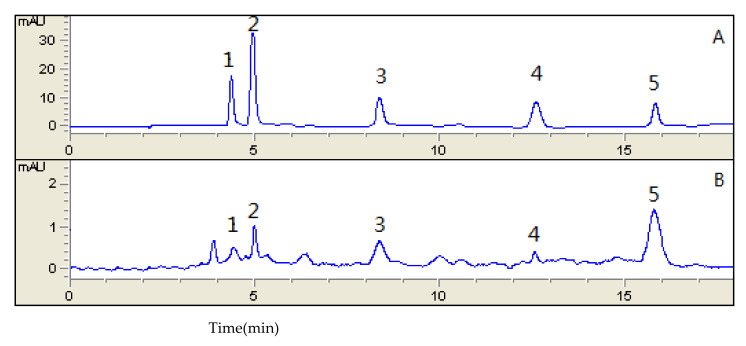
Chromatograms of a mix of standard carotenoids (**A**) and carotenoids extracted from cultivar ‘Jintong7’ (**B**). (1) Lutein, (2) Zeaxanthin, (3) β-Cryptoxanthin, (4) α-Carotene, and (5) β-Carotene.

As shown in Figure 2, we simultaneously analyzed the five main carotenoids (lutein, zeaxanthin, β-cryptoxanthin, α-carotene, and β-carotene) in each cultivar with HPLC.

The carotenoid content means of each variety are shown in Table S2. Normality analysis and calculations of variables related to the content of various carotenoids and total carotenoids (sum of the five carotenoids) in the yellow-flesh peaches were performed (Table 1). The Z-values of the one-sample Kolmogorov–Smirnov test for the five carotenoids all deviated from 1.0, and the asymptotic significance (two-sided) was less than 0.05, thus indicating that the content of the five carotenoids did not follow a normal distribution but instead showed skewed and concentrated distributions, with some extreme values reflecting the large variance and extreme deviations in the data. The Z-value of the total carotenoids was close to 1.0, and the asymptotic significance (two-sided) was greater than 0.05, thus indicating that the total carotenoids followed a normal distribution. The content of the five carotenoid fractions in the test resources varied considerably, with coefficients of variation ranging from 21.24% to 67.78%, and the variation amplitude and difference between Xmax-xmin were large. Overall, the five carotenoid content fractions ranged as follows: β-carotene (5.69 mg·kg^−1^ FW) > zeaxanthin (1.69 mg·kg^−1^ FW) > α-carotene (0.44 mg·kg^−1^ FW) > β-cryptoxanthin (0.41 mg·kg^−1^ FW) > lutein (0.26 mg·kg^−1^ FW) in descending order. The lutein content was very low, with the lowest coefficient of variation and amplitude of variation, ranging from 0.17 to 0.46 mg·kg^−^^1^ FW, with ‘B7029’ being the highest and ‘63-15-32’ being the lowest. The zeaxanthin content had a large coefficient of variation and amplitude of variation, ranging from 0.15 to 7.39 mg·kg^−^^1^ FW, with ‘Ruiguang2’ being the highest and ‘B7029’ being the lowest. Of note, the β-cryptoxanthin content was concentrated between 0.15 and 2.95 mg·kg^−^^1^ FW, but a few germplasms contained high levels of β-cryptoxanthin, such as ‘NJN76’ with a high content of 2.95 mg·kg^−^^1^ FW, followed by ‘TX4F244C’ with 2.41 mg·kg^−^^1^ FW. Thus, we observed large coefficients of variation, extreme differences, and slightly larger means. The α-carotene content was concentrated in the range of 0.14 to 1.75 mg·kg^−^^1^ FW, with ‘Dazhaohuangtao’ being the highest and ‘Habulite’ being the lowest. The distribution of the β-carotene content ranged from 0.93 to 16.71 mg·kg^−^^1^ FW, with ‘Jintong7’ being the highest and ‘77-26-7’ (16.04 mg·kg^−1^ FW) and ‘B7029’ being the lowest. The total carotenoid content was distributed in the range of 1.72 to 20.27 mg·kg^−^^1^ FW, with ‘Jintong7’ being the highest, ‘77-26-7’ and ‘77-20-5’ being lower, at 19.52 and 17.93 mg·kg^−^^1^ FW, respectively, and ‘Blazeprince’ being the lowest.

### 3.2. Comparison of Carotenoids across Fruit Developmental Periods

Correlation and variance analyses were performed to compare carotenoid concentrations among peach varieties with different fruit developmental periods (long (total 40), medium (total 59), and short (total 33)). The correlations between carotenoid concentration and fruit developmental periods are shown in Table 2. We observed positive correlations between the fruit developmental period and lutein and β-carotene. The correlation was not significant for lutein but was significant for β-carotene (*p* < 0.01). The fruit developmental period was significantly negatively correlated with zeaxanthin (*p* < 0.01) and β-cryptoxanthin (*p* < 0.01) and weakly negatively correlated with α-carotene and total carotenoids.

As shown in Table 2, we observed correlations among the content of the various carotenoids. A positive correlation was found between lutein and α-carotene; moreover, zeaxanthin was significantly positively correlated with β-cryptoxanthin (*p* < 0.01) and β-carotene (*p* < 0.01). Clear positive correlations between total carotenoids and both zeaxanthin (*p* < 0.01) and β-carotene (*p* < 0.01) were found.

According to the variance analysis of the types (short (total 40), middle (total 59), and long (total 33)) of fruit developmental periods (Table 3), the concentrations of lutein, α-carotene, and total carotenoids did not significantly differ among peach fruit in different developmental periods. However, the β-cryptoxanthin content was significantly lower in the fruit of peach cultivars with long rather than moderate or short fruit developmental periods (*p* < 0.05). Zeaxanthin was significantly lower in the fruit of peach cultivars with long rather than short fruit developmental periods (*p* < 0.05), whereas β-carotene was highest in the fruit of peach cultivars with long fruit developmental periods and significantly higher than short fruit developmental periods (*p* < 0.05). Thus, a longer fruit developmental period was associated with the greater β-carotene accumulation and lower zeaxanthin and β-cryptoxanthin accumulation.

### 3.3. Correlation between Carotenoids and Skin Type

A variance analysis was performed to compare the carotenoid concentrations in peach fruit with different fruit surface indumenta, including varieties with glabrous skin (total 44) and varieties with pubescent skin (total 88). As shown in Table 4, the concentrations of lutein, zeaxanthin, β-cryptoxanthin, and α-carotene in peach fruits with different fruit surface indumenta did not significantly differ, but significant differences in β-carotene and total carotenoids were observed among fruit with different surface types: the concentrations of β-carotene and total carotenoids were significantly greater in peach fruit with than without surface hairs (*p* < 0.05).

### 3.4. Comparison of Carotenoids in Yellow-Flesh Peach with Different Flesh Colors

A variance analysis comparing carotenoid concentrations in peach fruits with three flesh colors (yellow (total 34), golden yellow (total 66), and orange yellow (total 32)) revealed that the concentrations of lutein, β-cryptoxanthin, and α-carotene did not significantly differ (Table 5). The β-cryptoxanthin content in the fruit of golden yellow peach cultivars was slightly higher than that in the fruit of yellow and orange yellow peach cultivars, and the content of lutein and α-carotene was similar in all three flesh colors. Zeaxanthin in the fruit of yellow peach cultivars was significantly lower than in the fruit of golden yellow and orange yellow peach cultivars (*p* < 0.05). Significant differences in β-carotene and total carotenoids were found among the three flesh colors. Thus, the concentrations of β-carotene and total carotenoids significantly increased as the flesh color deepened.

## 4. Discussion

Yellow-flesh peaches are rich in carotenoids and thus have potential for the development of healthy functional products. The main carotenoid components in yellow flesh peaches include lutein, zeaxanthin, β-cryptoxanthin, α-carotene, and β-carotene [19]. Herein, the total carotenoid content of 132 yellow-fleshed peaches was analyzed and found to range from 1.72 to 20.27 mg·kg^−1^, and the levels of the carotenoid compounds, in descending order, were β-carotene, zeaxanthin, α-carotene, β-cryptoxanthin, and lutein, in agreement with findings reported by Yan et al. [15]. In the carotenoid biosynthetic pathway in plants, lycopene is catalytically cyclized to β-carotene and α-carotene. β-Carotene is hydroxylated to produce β-cryptoxanthin, and β-cryptoxanthin is converted to zeaxanthin by the action of specific enzymes. α-Carotene is altered into lutein by successive hydroxylation reactions [31]. Thus, the five carotenoids show interconversion, and the components are closely interrelated: changes in the content of a component may lead to significant changes in the content of other components. Our results explain the highly significant correlations among the various carotenoids, the variations in the content of the five carotenoids, their instability, the absence of a normal distribution, and the existence of extreme values, as well as other phenomena. The indicator of total carotenoids followed a normal distribution. Therefore, screening yellow flesh peach resources for germplasms with high total carotenoid and high individual carotenoid content is theoretically and practically feasible. Using HPLC, Polish researchers evaluated 20 peach varieties and screened for medium pits, high dry matter, total sugar content, and high carotenoid content in ‘Harrow beauty’, ‘Kijowska wczesna’, and ‘Jersey land’ to make healthful dry snacks [30]. Herein, we identified high levels of zeaxanthin in ‘Ruiguang2’, β-cryptoxanthin in ‘NJN76’ and ‘TX4F244C’, and β-carotene and total carotenoids in ‘Jintong7’, ‘77-26-7’, and ‘77-20-5’. The results may provide a basis for the rational and effective use of yellow flesh peach resources.

Studying the relationships between color and carotenoids in fruits and vegetables is especially challenging because it involves perceived quality and consumers’ choices, as well as the nutritional value. Even in one species, different colors have markedly different carotenoid profiles. In paprika, capsanthin and capsorubin are the main carotenoids in red paprika, lutein is abundant in yellow and green paprika, and β-carotene is present in orange paprika [32,33]. The traditional yellowish or orange color of ripe sweet oranges is mostly due to carotenoid pigments, red-fleshed mutant colors arise from the carotenoid lycopene [34], and the hues of most orange juices range from yellow to orange and are mainly due to their carotenoid content [35,36]. Xiong et al. [37] focused on the changes in carotenoid fractions and contents in loquat flesh during the coloring period. They suggested that the β-carotene content was the main factor influencing the accumulation of total carotenoids and the differences in flesh color among varieties. Similarly, Yang’s research [38] indicated that β-carotene is a major carotenoid component affecting carotenoid accumulation and color levels (deep yellow and light yellow) in the flesh of two pineapple cultivars. This research showed significantly elevated total β-carotene and carotenoids in peach with deeper flesh colors. The significant increases in the total β-carotene and carotenoids occurred in peach having deeper flesh colors. A more pronounced orange color was observed because β-carotene was the predominant component. Orange-yellow flesh, compared with the other flesh colors, contained nearly twice the amount of β-carotene. Thus, the differences in peach flesh color may be due to differences in the β-carotene content, in agreement with the results of studies in loquat and pineapple, as well as those reported by Yan. [39] in peaches. However, Yan. [40] also indicated that the color associated with β-cryptoxanthin tends to be orange-yellow, whereas that associated with α-carotene tends to be light yellow. These patterns were not found in the 132 yellow-fleshed peaches in this study, probably because of the large sample size. We found that the zeaxanthin content increased as the flesh color deepened, thus indicating that zeaxanthin also contributes to the differences in flesh color. The lutein content significantly differed between golden and orange flesh colors, thereby suggesting that lutein contributed to the difference in only this color range. However, we have to point out here that lutein, as one type of Xanthophylls, accumulates in yellow peach as esters [41], meaning the individual molecules are covalently bonded to each other with ester bonds. To separate them into free xanthophylls that can be identified by HPLC, the ester bonds must be broken. This is usually achieved by a chemical reaction named saponification. Based on this research’s method without saponification, only carotenes (non-oxygenated carotenoids) can be separated and identified by HPLC, and this is probably the reason why, in this report, lutein is the lowest content carotenoid in the samples.

The relationships between the glabrous and pubescent skins and the content of carotenoid fractions in yellow-fleshed peaches are relatively novel results. Both β-carotene and total carotenoids were significantly higher in fruit with pubescent than glabrous surfaces. Studies on citrus have shown that carotenoids in the peel are an important stock in the pulp and that light conditions significantly affect the accumulation of carotenoids in the peel [40]. In addition, in a study of cantaloupe [42], fruit color presentation was found to be strongly influenced by direct and diffuse light. Most of the fruit surface is exposed to direct light, which tends to increase fruit temperature and inhibit the synthesis of yellow carotenoids, whereas scattered light favors the synthesis of yellow carotenoids. Back-shaded fruits are the first to show the yellow color associated with carotenoids because they receive more scattered light, thus resulting in greater coloration in fruit that is covered by leaves. In yellow-fleshed peaches, Dong et al. [23] suggested that the light environment influences carotenoid synthesis. Light can produce differences in carotenoid composition and content between bagged and unbagged fruit by regulating the conversion of chloroplasts to colored bodies and the expression of HYB genes during yellow-fleshed peach fruit development. Yan et al. [43] also found that different illumination levels and spectral treatments affect the accumulation of lutein and β-carotene in peach fruit flesh during ripening. This study found that the concentrations of β-carotene and total carotenoids in peach fruit with surface hairs were significantly greater than those in fruit without surface hairs (*p* < 0.05). The presence or absence of peach surface hairs might possibly have caused differences in light and temperature conditions between the skin and flesh in the form of microenvironmental signals, thereby directly or indirectly influencing the accumulation of carotenoids in the flesh. The basis for this finding must be further investigated in detail. The density and length of pubescent surfaces should be evaluated, and the carotenoid content of the skin and flesh, as well as the expression levels of the relevant synthetic genes, should be measured and compared.

We found that longer developmental periods were associated with greater accumulation of lutein and β-carotene and lower accumulation of zeaxanthin and β-cryptoxanthin. The differences in total α-carotene and carotenoids were not significant. The differences in the total carotenoid content and the content of each component in yellow peach might have been due to differences in the environmental growth conditions, such as temperature and light, caused by differences in the length of the reproductive period. The underlying mechanisms should be investigated in further comparative studies.

## 5. Conclusions

There were large differences in the carotenoid contents among yellow-flesh peach germplasms. The carotenoids contents were, from high to low, as follows: β-carotene > zeaxanthin > α-carotene > β-cryptoxanthin > lutein. The carotenoid contents were correlated with fruit developmental period, flesh color, and fruit surface indumenta. The longer the fruit’s developmental period, the greater the β-carotene accumulation; however, the lower were the zeaxanthin and β-cryptoxanthin accumulations. The zeaxanthin, β-carotene, and total carotenoids concentrations significantly increased as the flesh color deepened, but the lutein and α-carotene levels remained similar among the three flesh colors. β-carotene and total content characterized by pubescent > glabrous skin. This study screened some varieties having high carotenoid contents, including zeaxanthin in ‘Ruiguang2’, β-cryptoxanthin in ‘NJN76’ and ‘TX4F244C’, and β-carotene and total carotenoids in ‘Jintong7’, ‘77-26-7’, and ‘77-20-5’. The results will help researchers and breeders understand carotenoid accumulation character and select new competitive peach varieties with high carotenoid contents.

## Figures and Tables

**Table 1:** Variations in the carotenoid content of yellow-flesh peach.

Index	Lutein	Zeaxanthin	β-Cryptoxanthin	α-Carotene	β-Carotene	Total Content
Mean	0.26	1.69	0.41	0.44	5.69	8.55
Standard deviation	0.06	1.15	0.32	0.24	2.73	3.42
Variation amplitude	0.17–0.46	0.15–7.39	0.15–2.95	0.14–1.75	0.93–16.71	1.72–20.27
Xmax-xmin	0.29	7.23	2.80	1.61	15.79	18.56
Variable coefficient (%)	21.24	68.09	67.78	55.29	48.01	39.96
Kolmogorov–Smirnov	1.53	1.74	3.01	1.38	1.48	1.07
Asymptotic sig. (two-sided)	0.02	0.01	0.00	0.04	0.03	0.20

Note: Carotenoid content’s unit is mg·kg^−1^ FW. Data show the mean of three replicates from samples at harvest in 2019.

**Table 2:** Correlation analyses between carotenoids and fruit developmental periods and among carotenoid concentrations.

Correlation Coefficient	Fruit Development Period	Lutein	Zeaxanthin	β-Cryptoxanthin	α-Carotene	β-Carotene	Total Carotenoids
Fruit development period	1.000						
Lutein	0.134	1.000					
Zeaxanthin	−0.447 _**_	−0.074	1.000				
β-cryptoxanthin	−0.236 _**_	0.051	0.529 _**_	1.000			
α-carotene	−0.103	0.431 _**_	0.081	0.066	1.000		
β-carotene Total carotenoids	0.335 _**_ −0.071	−0.110 −0.082	0.262 _**_ 0.600 _**_	0.168 0.211	0.154 0.205	1.000 0.922_**_	1.000

Note: _**_ Significant difference at the 0.01 level (*p* < 0.01).

**Table 3:** Carotenoid concentrations of yellow-flesh peach with different fruit developmental periods.

Carotenoid	Fruit Development Period (d)		
	Short	Middle	Long
Lutein	0.25 ± 0.05 ^a^	0.26 ± 0.05 ^a^	0.28 ± 0.06 ^a^
Zeaxanthin	2.25 ± 1.37 ^a^	1.81 ± 1.15 ^ab^	1.10 ± 0.58 ^b^
β-cryptoxanthin	0.56 ± 0.46 ^a^	0.49 ± 0.31 ^a^	0.38 ± 0.1 ^b^
α-carotene	0.45 ± 0.26 ^a^	0.44 ± 0.28 ^a^	0.42 ± 0.17 ^a^
β-carotene	4.91 ± 1.94 ^b^	5.78 ± 2.84 ^a^	6.18 ± 3.01 ^a^
Total carotenoids	8.44 ± 2.97 ^a^	8.76 ± 3.61 ^a^	8.36 ± 3.53 ^a^

Note: Data showed the mean from samples in 2019. Different letters indicate significant differences at *p* < 0.05.

**Table 4:** Comparison of carotenoid content of yellow-flesh peach with different fruit surface indumenta.

Carotenoid	Surface Indumentum	
	Glabrous Skin	Pubescent Skin
Lutein	0.25 ± 0.05 ^a^	0.27 ± 0.06 ^a^
Zeaxanthin	1.83 ± 1.4 ^a^	1.62 ± 1.01 ^a^
β-cryptoxanthin	0.52 ± 0.43 ^a^	0.45 ± 0.25 ^a^
α-carotene	0.41 ± 0.26 ^a^	0.45 ± 0.23 ^a^
β-carotene	4.66 ± 1.44 ^b^	7.19 ± 2.06 ^a^
Total carotenoids	7.67 ± 2.78 ^b^	9.97 ± 2.62 ^a^

Note: Data showed the mean from samples in 2019. Different letters indicate significant differences at *p* < 0.05.

**Table 5:** Comparison of carotenoid content of yellow-flesh peach with different flesh colors.

Carotenoid	Flesh Color		
	Yellow	Golden	Orange
Lutein	0.26 ± 0.05 ^a^	0.27 ± 0.06 ^a^	0.25 ± 0.03 ^a^
Zeaxanthin	1.38 ± 0.52 ^b^	1.79 ± 0.45 ^a^	1.83 ± 0.57 ^a^
β-Cryptoxanthin	0.42 ± 0.21 ^a^	0.51 ± 0.27 ^a^	0.45 ± 0.11 ^a^
α-Carotene	0.44 ± 0.27 ^a^	0.43 ± 0.26 ^a^	0.45 ± 0.16 ^a^
β-Carotene	4.35 ± 0.89 ^c^	5.31 ± 1.21 ^b^	8.19 ± 2.62 ^a^
Total carotenoids	6.45 ± 1.89 ^c^	8.30 ± 2.20 ^b^	11.17 ± 3.02 ^a^

Note: Data show the mean from samples in 2019. Different letters indicate significant differences at *p* < 0.05.

## Data Availability

Data are contained within the article.

## Supplementary Materials

The following are available online at https://www.mdpi.com/article/10.3390/foods11121669/s1, Table S1: Descriptors of yellow-flesh peach. Table S2: Carotenoid content of yellow-flesh peach.
