# Supplementary material for: Carotenoid Profiling of Yellow-Flesh Peach Fruit
Source: Foods. 2022 Jun 7;11(12):1669. doi: 10.3390/foods11121669 (PMC9222759; doi:10.3390/foods11121669)
Supplement: Supplementary file 1 [file foods-11-01669-s001.zip › foods-1720973-supplementary.pdf]

Supplementary Tables

Table S1 Descriptors of yellow-flesh peach

Peach varieties name	Fruit development period (d)	Surface indumentum	Flesh color	Peach varieties name	Fruit development period (d)	Surface indumentum	Flesh color	Peach varieties name	Fruit development period (d)	Surface indumentum	Flesh color
Chaowuyuehuo	63	GS	Y	Huyou003	97	PS	G	Xiyanghuangrou	120	GS	G
Springbaby	63	PS	Y	Zaohuangpantao	97	GS	G	Shasita	121	GS	O
Zaofengtian	63	PS	Y	Tubade	98	GS	G	Chunguan	122	GS	G
Shuguang	68	PS	Y	Honggang	101	GS	O	Taiyangnianhe	122	GS	G
Wuyuehuo	70	PS	Y	FlavorGold	102	GS	Y	Zaoliguangtao	122	PS	Y
Jinshanzaohong	74	PS	Y	TX4F244C	102	GS	O	Guantao14	124	GS	O
Zaohongbaoshi	79	PS	Y	Hongding	102	GS	Y	Ruiguang20	124	PS	O
Armking	80	PS	O	Pegaso	103	PS	G	Huanxiang	125	PS	G
Zijinhong1	80	PS	Y	Sunblaze	103	PS	G	Buleidingfeng	126	GS	Y
Quetta	81	PS	Y	Hagen	103	GS	G	Dikesong	126	GS	G
Jinhualu	81	GS	G	Dazhaohuangtao	104	GS	G	Veteran	127	GS	O
Ruiguang2	81	PS	Y	Kalinan	104	GS	O	85-22-35	128	GS	G
63-15-32	83	GS	Y	Weisuweiao	105	GS	Y	Huangroupantao	128	GS	G
63-15-75	83	GS	G	Hongyoutao4	106	PS	Y	Jinyan	128	GS	O
Crimsonbaby	83	PS	Y	Mingxing	106	GS	O	Jintong7	129	GS	O
85-13-24	85	GS	G	Shuangfo	106	PS	Y	Ligelante	129	PS	Y
Shuangxihong	85	PS	Y	Guantao5	107	GS	O	77-18-1	131	GS	O
Flordaking	86	GS	Y	NJN78	108	PS	G	Dahuaaibaotai	131	GS	G
TX2B7N	86	PS	Y	Jinhui	108	GS	G	61-299	133	GS	O
NJN70	87	PS	G	Nectaross	109	PS	G	Feilipu	133	GS	O

NJN76	87	PS	G	Duli	109	PS	Y	63-3-9	134	GS	G
Sunsplash	87	PS	G	5-24-18	110	GS	G	Flameprince	134	GS	Y
Kaixuan	88	GS	G	Resino	110	GS	G	Jinfeng	135	GS	G
TX2C104N	89	PS	Y	Zhejin3	110	GS	G	Jinluoman	136	GS	G
Changlihuangrou	89	GS	G	Blazeprince	111	GS	Y	Tianjinhuangrou	136	GS	G
Chengxiang	89	GS	O	Jintong5	112	GS	O	63-9-26	137	GS	G
Flordacrest	90	GS	G	Lianhuang	112	GS	O	Huangjintao	137	GS	Y
NJN72	90	PS	Y	SunKing	113	PS	Y	81-22-71	138	GS	G
RedJune	90	PS	G	StarkDelicious	114	PS	G	Deguohuangjin	138	GS	G
Sunraycer	90	PS	G	Huangjinmeili	114	GS	O	Xiaohuaaibaotai	138	GS	G
Ao19	90	PS	G	Kashenliguang	114	PS	O	NF9260	139	PS	G
jinmali	90	GS	G	77-20-5	115	GS	O	Qiukui	140	GS	G
Ruiguang22	90	PS	Y	77-26-7	115	GS	O	77-15-2	141	GS	G
801	91	PS	G	Sungrand	115	PS	G	77-26-33	141	GS	G
85-13-19	91	GS	G	Habulite	117	GS	G	78-19-40	142	GS	G
5-8-23	93	GS	G	Luomianna	117	GS	G	Huolianjindan	142	GS	G
Tropicprince	94	GS	O	82-61-40	118	GS	O	Jincheng	142	GS	O
TX4C189LN	94	PS	Y	Heyangyoutao	118	PS	Y	Yangtao40	142	GS	G
Fafulaite3	94	GS	O	B7029	119	GS	G	78-19-16	143	GS	G
Zaohong2	94	PS	O	81-14-63	120	GS	O	78-20-17	145	GS	G
Jinxu	95	GS	G	81-20-16	120	GS	G	Juhuang	145	GS	O
Zijinhong2	95	PS	G	Faye	120	GS	G	Jintong8	146	GS	O
Zaojinlu	96	GS	G	Jintong6	120	GS	O	Hafu	147	GS	Y
Beibeile	97	GS	O	Jinying	120	GS	G	Shamenlaite	151	GS	G

Note: GS: glabrous skin; PS: pubescent skin; Y:yellow; G: golden; O: orange

Table S2 Carotenoid content of yellow-flesh peach

Peach varieties name	Lutein	Zeaxanthin	β-cryptoxanthin	α-carotene	β-carotene	Total content	Peach varieties name	Lutein	Zeaxanthin	β-cryptoxanthin	α-carotene	β-carotene	Total content
Chaowuyuehuo	0.27	1.15	0.34	0.47	2.69	4.92	Resino	0.21	1.72	0.37	0.48	6.62	9.40
Springbaby	0.27	2.10	0.39	0.41	3.79	6.96	Zhejin3	0.30	1.69	0.45	0.15	5.22	7.81
Zaofengtian	0.22	1.35	0.35	0.15	2.71	4.78	Blazeprince	0.17	0.24	0.15	0.15	1.01	1.72
Shuguang	0.34	1.97	0.43	0.66	4.42	7.82	Jintong5	0.26	1.91	0.45	0.49	6.82	9.94
Wuyuehuo	0.30	1.29	0.46	0.48	4.98	7.51	Lianhuang	0.22	1.72	0.41	0.42	7.17	9.94
Jinshanzaohong	0.40	2.61	0.49	0.79	3.86	8.14	SunKing	0.23	0.71	0.32	0.16	2.57	3.99
Zaohongbaoshi	0.43	3.54	0.50	0.80	5.01	10.28	StarkDelicious	0.23	1.04	0.37	0.50	4.42	6.56
Armking	0.33	1.25	0.46	0.47	5.29	7.81	Huangjinmeili	0.25	2.07	0.52	0.64	9.70	13.20
Zijinhong1	0.26	1.22	0.45	0.16	3.72	5.81	Kashenliguang	0.19	0.31	0.22	0.14	1.71	2.57
Quetta	0.20	3.46	0.44	0.16	5.59	9.85	77-20-5	0.22	2.85	0.55	0.40	13.93	17.93
Jinhualu	0.24	1.33	0.51	0.52	4.43	7.03	77-26-7	0.20	2.23	0.57	0.48	16.04	19.52
Ruiguang2	0.26	7.39	1.24	0.65	7.11	16.64	Sungrand	0.21	0.81	0.35	0.15	4.59	6.11
63-15-32	0.17	2.43	0.53	1.01	6.02	10.17	Habulite	0.17	1.09	0.32	0.14	3.98	5.70
63-15-75	0.24	2.67	0.46	0.66	4.01	8.04	Luomianna	0.26	2.08	0.52	0.58	5.93	9.37
Crimsonbaby	0.25	1.30	0.35	0.43	3.82	6.15	82-61-40	0.23	1.91	0.41	0.69	10.06	13.31
85-13-24	0.26	2.89	0.69	0.55	4.40	8.81	Heyangyoutao	0.24	0.74	0.42	0.16	5.16	6.73
Shuangxihong	0.24	0.52	0.61	1.16	3.96	6.49	B7029	0.46	0.15	0.28	0.15	0.93	1.97
Flordaking	0.28	0.71	0.40	0.63	6.66	8.67	81-14-63	0.26	1.10	0.44	0.56	7.73	10.10
TX2B7N	0.25	1.11	0.38	0.43	4.44	6.62	81-20-16	0.25	1.03	0.35	0.57	5.48	7.69
NJN70	0.24	2.86	0.43	0.30	5.41	9.23	Faye	0.24	1.22	0.36	0.55	5.11	7.48
NJN76	0.26	4.56	2.95	0.15	4.01	11.93	Jintong6	0.26	3.35	0.75	0.54	8.74	13.63
Sunsplash	0.31	2.71	0.55	0.54	6.84	10.94	Jinying	0.27	0.82	0.32	0.51	4.33	6.25
Kaixuan	0.24	2.27	0.57	0.40	12.37	15.85	Xiyanghuangro	0.21	0.81	0.31	0.34	4.68	6.35

							u						
TX2C104N	0.23	2.26	0.43	0.15	5.46	8.53	Shasita	0.22	1.91	0.35	0.53	11.02	14.02
Changlihuangrou	0.23	2.57	0.55	0.34	7.12	10.80	Chunguan	0.27	2.54	0.37	0.43	5.34	8.96
Chengxiang	0.23	3.22	0.46	0.15	4.57	8.63	Taiyangnianhe	0.22	1.43	0.39	0.47	8.52	11.03
Flordacrest	0.23	0.88	0.37	0.39	3.56	5.44	Zaoliguangtao	0.24	0.50	0.34	0.16	4.84	6.07
NJN72	0.28	0.22	0.15	0.35	2.00	2.99	Guantao14	0.27	1.48	0.35	0.42	9.81	12.34
RedJune	0.23	2.31	0.40	0.15	3.99	7.08	Ruiguang20	0.22	1.43	0.74	0.65	7.47	10.51
Sunraycer	0.26	2.14	0.46	0.16	4.35	7.37	Huanxiang	0.25	1.65	0.44	0.37	5.96	8.67
Ao19	0.21	1.28	0.43	0.15	3.47	5.54	Buleidingfeng	0.25	0.36	0.16	0.16	1.80	2.74
jinmali	0.29	3.39	0.39	0.35	3.61	8.03	Dikesong	0.27	2.51	0.39	0.39	11.36	14.92
Ruiguang22	0.25	3.36	0.69	0.72	8.49	13.51	Veteran	0.23	2.36	0.45	0.41	9.84	13.28
801	0.23	4.97	1.08	0.48	5.94	12.69	85-22-35	0.24	1.28	0.53	0.43	6.67	9.15
85-13-19	0.32	3.89	1.08	0.15	7.07	12.50	Huangroupantao	0.38	0.57	0.48	0.74	6.40	8.57
5-8-23	0.30	5.32	0.66	0.38	6.36	13.02	Jinyan	0.22	1.35	0.43	0.33	6.44	8.76
Tropicprince	0.21	1.29	0.36	0.45	4.58	6.89	Jintong7	0.26	1.89	0.64	0.78	16.71	20.27
TX4C189LN	0.27	2.14	0.53	0.92	5.69	9.56	Ligelante	0.20	0.32	0.15	0.15	2.45	3.27
Fafulaite3	0.29	2.58	0.47	0.75	6.83	10.90	77-18-1	0.30	0.94	0.37	0.55	10.04	12.21
Zaohong2	0.27	1.82	0.42	0.32	4.72	7.55	Dahuaaibaotai	0.20	0.60	0.30	0.35	3.79	5.24
Jinxu	0.27	2.44	0.47	0.70	7.45	11.33	61-299	0.24	1.22	0.38	0.50	7.84	10.18
Zijinhong2	0.23	2.73	0.40	0.69	4.07	8.12	Feilipu	0.27	0.93	0.39	0.47	11.48	13.54
Zaojinlu	0.27	3.70	0.42	0.14	3.81	8.34	63-3-9	0.30	1.36	0.37	0.41	6.00	8.44
Beibeile	0.19	4.38	0.48	0.15	4.87	10.07	Flameprince	0.20	0.66	0.34	0.43	4.13	5.77
Huyou003	0.18	0.86	0.67	0.94	6.65	9.30	Jinfeng	0.32	0.48	0.33	0.39	4.13	5.65
Zaohuangpantao	0.30	2.83	0.59	0.94	4.25	8.91	Jinluoman	0.25	1.25	0.38	0.53	5.37	7.79

Tubade	0.24	1.66	0.35	0.64	12.36	15.24	Tianjinhuangrou	0.45	0.50	0.37	0.58	4.52	6.42
Honggang	0.20	2.18	0.38	0.42	4.93	8.11	63-9-26	0.24	0.77	0.33	0.43	5.74	7.52
FlavorGold	0.24	2.18	0.81	0.50	3.25	6.98	Huangjintao	0.38	0.45	0.40	0.48	4.30	6.00
TX4F244C	0.31	2.72	2.41	0.31	7.81	13.57	81-22-71	0.22	1.10	0.43	0.55	7.45	9.75
Hongding	0.22	0.34	0.39	0.38	4.43	5.76	Deguohuangjin	0.28	0.62	0.36	0.44	4.06	5.74
Pegaso	0.21	1.79	0.45	0.19	3.84	6.47	Xiaohuaaibaotai	0.32	0.48	0.29	0.15	3.51	4.74
Sunblaze	0.32	1.15	0.43	0.52	3.88	6.30	NF9260	0.36	1.23	0.43	0.42	6.59	9.03
Hagen	0.23	2.12	0.43	0.52	6.31	9.61	Qiukui	0.23	0.83	0.31	0.15	5.32	6.85
Dazhaohuangtao	0.30	1.13	0.56	1.75	6.14	9.87	77-15-2	0.34	0.68	0.35	0.16	4.12	5.66
Kalinan	0.23	0.97	0.33	0.40	3.65	5.58	77-26-33	0.26	1.23	0.37	0.35	4.58	6.80
Weisuweiao	0.26	1.07	0.35	0.47	4.89	7.03	78-19-40	0.44	0.65	0.34	0.18	3.92	5.53
Hongyoutao4	0.25	0.62	0.36	0.42	5.83	7.47	Huolianjindan	0.28	1.04	0.28	0.26	3.45	5.31
Mingxing	0.25	2.22	0.47	0.15	6.37	9.46	Jincheng	0.27	0.76	0.35	0.36	3.33	5.07
Shuangfo	0.19	1.10	0.38	0.16	5.07	6.90	Yangtao40	0.36	0.62	0.40	0.63	4.35	6.35
Guantao5	0.29	2.43	0.49	0.45	10.69	14.35	78-19-16	0.34	1.17	0.44	0.65	5.03	7.63
NJN78	0.23	1.30	0.41	0.14	6.04	8.12	78-20-17	0.36	0.59	0.35	0.15	4.13	5.57
Jinhui	0.30	1.22	0.37	0.39	1.90	4.18	Juhuang	0.25	1.80	0.44	0.49	10.36	13.34
Nectaross	0.18	1.25	0.36	0.28	4.38	6.45	Jintong8	0.28	1.11	0.37	0.68	6.31	8.74
Duli	0.25	0.31	0.31	0.30	3.72	4.89	Hafu	0.31	0.95	0.33	0.47	4.42	6.49
5-24-18	0.21	3.16	0.33	0.39	3.76	7.84	Shamenlaite	0.22	1.79	0.32	0.42	2.66	5.40

Note: Data showed the mean of three replicates from samples at harvest in 2019. Carotenoid content's unit is mg·kg^{-1} FW.
